# Traditional farmers’ pig trait preferences and awareness levels toward reproductive biotechnology application in Zambia

**DOI:** 10.5455/javar.2022.i591

**Published:** 2022-06-26

**Authors:** Rubaijaniza Abigaba, Pharaoh Collins Sianangama, Progress H. Nyanga, Wilson N. M. Mwenya, Edwell S. Mwaanga

**Affiliations:** 1 Department of Animal Science, University of Zambia, Lusaka, Zambia; 2 Department of Biomedical Sciences, University of Zambia, Lusaka, Zambia; 3 Department of Geography and Environmental Studies, University of Zambia, Lusaka, Zambia

**Keywords:** Awareness, indigenous pig trait, pig production, reproductive, biotechnology, traditional farmers

## Abstract

**Objective:**

This study assessed traditional farmers’ preferences for indigenous pigs and their awareness levels toward reproductive biotechnology.

**Materials and Methods:**

This cross-sectional, descriptive study employed a mixed-methods concurrent triangulation design with a pragmatic approach. For quantitative data collection, a randomly selected sample size of 622 respondents was interviewed using a structured questionnaire. A semi-structured interview guide was used in seven focus group discussions (FGDs) for qualitative data. For quantitative data, descriptive statistics were used to find out how often something happened, and chi-square tests were used to look for relationships. For qualitative data, thematic analysis was used.

**Results:**

The majority (66.9%) of respondents were female, and they had largely (64.8%) attended a primary level of education. A slight majority (43.1%) of the respondents were 30–45 years old. Most respondents (65.1%) were low-income earners (below K500). Incidentally, the majority (74.1%) of respondents had low awareness of reproductive biotechnologies. Respondents’ awareness levels were associated with gender (*p* < 0.001), education (*p* < 0.001), income (*p* < 0.01), and not age (p > 0.05). With regard to trait preference, a total of seven indigenous pig traits were reportedly preferred, of which disease resistance (25.9%) and litter size (23.8%) were the most preferred. From FGDs, participants valued indigenous pigs, citing many preferred traits, of which disease resistance and litter size were the most emphasized traits. Some participants were aware of many reproductive biotechnologies and their perceived advantages. However, they were more familiar with artificial insemination, heat detection methods, and synchronization.

**Conclusions:**

The majority of respondents had low awareness of reproductive biotechnologies. Respondents’ awareness was associated with sociodemographic characteristics. The identified indigenous pig traits preferred by traditional farmers included adaptive and productive traits.

## Introduction

Zambia is among the countries with many livestock species [1,2]. However, the vast majority of farmers (>80%) are stuck in low-productive traditional farming, devoid of methods and strategies to achieve full productivity [2–4]. The food demand–supply gap characterized by sustained food and nutritional insecurity among the rural population remains unresolved; over 40% of the rural population lacks access to adequate food, and 35% of the children are stunted [5,6]. Moreover, the demand for food, especially meat, is expected to triple by 2050 as the human population increases [7]. Although policies that promote diversification to include livestock production, improve production through biotechnology, and conserve indigenous genetic resources have been enacted, agricultural production diversification remains low [4–7]. There is a need for more efficient food production systems to reduce the food demand–supply gap; of note, pig rearing was recommended as a strategy to minimize the existing animal protein deficit [2,8,9].

Despite the previous recommendation, Zambian pig production and pork availability remain low; for example, the total national flock and available pork in 2020 were 1.16 million and 65,244 metric tons, respectively [8,10]. Both exotic and indigenous pigs are reared in Zambia; however, the latter, including Lusitu and Nsenga breeds, constitute 65% of the national flock [4,11]. Even though their productivity is low, traditional farmers still value indigenous pigs [8], also confirmed by the 65% proportion; thus, there is a need to identify the possible traits contributing to the value farmers attach to Lusitu and Nsenga pigs. There is scanty information about the adaptability of indigenous pigs to a range of harsh rearing conditions. Nevertheless, suggestions were made for increased production and productivity [4,12]. One of the recommended means for increased production is the application of reproductive biotechnologies such as artificial insemination (AI) and AI-supporting biotechnologies [8,13,14]. They improve reproductive efficiency, the backbone of animal production and productivity [13,15]. However, reproductive biotechnology applications in Zambia are limited or not used by traditional pig farmers [4,8]; therefore, there is an urgent need to promote their utilization.

The Zambian government wishes to modernize production; however, widespread biotechnology application necessitates a better understanding of psycho-socioeconomic factors, such as awareness, attitudes, the extension system, and biotechnology-related factors [16,17]. Biotechnology awareness is one of the crucial factors since it facilitates the prediction of reproductive biotechnology acceptance and/or adoption rate(s) [16]. This is because learning new biotechnology starts with becoming aware of it and understanding its relative advantages. A person forms attitudes toward it and thereby derives social and personal choices like biotechnology adoption [16,18]. However, there is a lack of information on farmers’ biotechnology awareness, which would affect the prediction of acceptance and adoption rates and thus lead to ineffective policy formulation and implementation. Furthermore, the government must use responsive approaches to the farmers’ needs and choices to achieve their production objectives [19,20]. Since the Lusitu and Nsenga pigs are mainly reared and possibly suitable under the local rearing conditions, there is a need to ascertain the various traits that farmers cherish to inform biotechnology policy and further research appropriately.

This study aimed to generate crucial information about reproductive biotechnology awareness and pig trait preferences; the objectives were (1) to ascertain the awareness levels of traditional pig farmers toward reproductive biotechnology application; (2) to determine the relationship between farmers’ biotechnology awareness levels and their sociodemographic characteristics; and (3) to ascertain traditional farmers’ evaluation of indigenous pigs and traits of their preference.

## Methods and Materials

### Ethical clearance and informed consent

The Biomedical Research Ethics Committee of the University of Zambia approved this study (No. 1595-2021). All participants were told what the goals of the study were, and they all signed off on the study.

### Study area and period

This study was conducted in Petauke and Gwembe districts of the Eastern and Southern provinces, respectively, from February to September 2021. Petauke lies within the agro-ecological region II, while Gwembe lies in the agro-ecological region I. Petauke and Gwembe districts have rain patterns and temperatures of 750–1,000 mm; 30°C–32°C and 400–750 mm; 30°C–36°C, respectively [4]. The study areas had the highest pig proportion of the national flock [1]. Most (65%) of the pigs reared are indigenous, including Nsenga and Lusitu pigs; Petauke and Gwembe are the places of origin for Nsenga and Lusitu pigs, respectively. The rest of the pigs raised are exotic, namely Large White, Landrace, and Duroc breeds [11]. Furthermore, the traditional farming system dominates pig production in Zambia (90%) [4].

### Study design

This study was a cross-sectional, descriptive survey that employed a mixed-methods concurrent triangulation design with a pragmatic epistemological approach to allow for intersubjectivity through complementarity and confirmation of findings from the quantitative and qualitative study phases. It integrated the philosophical frameworks of both postpositivism and interpretivism, interweaving qualitative and quantitative data to explain the subject matter meaningfully. The data were collected using both quantitative and qualitative data collection methods, with a particular emphasis on psycho-socioeconomic issues, such as farmers’ cherished indigenous pig traits and related key sociological aspects of pig farming [21,22]. A qualitative–quantitative methodological triangulation was used to collect data and figure out what it meant. The goal was to get a deep and wide understanding of the subject [23,24].

### Sampling and data collection

For the quantitative phase or questionnaire survey, a multistage purposeful random sampling technique was used to recruit traditional farmers for data collection [23], and the selection criteria were appropriate and sensitive to the possible study bias for result validity. The data were collected using a well-designed questionnaire based on previous research and modified to fit the study objectives [25]. This tool included 12 dichotomous questions on biotechnology awareness and other dichotomous, multiple-choice, open-ended questions on trait preference and demographic aspects. A pretest was conducted for tool validation, including internal consistency for the awareness scale (KR-20 = 0.790). 

The sample size used was estimated using the following formula applicable at different population proportion levels and confidence levels [26]: 


$$n=\frac{N}{1+N{ɛ}^{2}}$$

where, n = minimum sample size,* N* = population size, ɛ = adjusted margin of error $\left[ɛ=\frac{\rho e}{t}\right]$, e = degree of accuracy expressed as proportion (margin of error at 0.05), ρ =number of standard deviations = 2 (dichotomous responses), and *t* = *t*-value for the selected alpha level or confidence level = 1.96 at 95% confidence interval [26,27]. The formula yielded a value of 383; however, an adjustment of 62% was made to compensate for sampling errors due to a mix of methods used in multistage sampling. The final sample size for the interviewer–administered questionnaire survey was 622 farmers, of which 353 and 269 farmers were recruited from Petauke and Gwembe districts, respectively. They were randomly selected from 9 agricultural camps, with 6 villages selected per camp; 5–20 respondents were randomly recruited from each village.

For the qualitative phase, a criterion-i sampling strategy was used to recruit participants [23]. In this case, participants were selected from those who had participated in the quantitative phase of this study. The focus group discussion (FGD) interview guide used for the interviews comprised of open-ended questions with a focus on biotechnology awareness and farming characteristics. Seven FGDs, three for females and four for males, were conducted; the number of participants per FGD ranged from 6 to 9. The procedures for FGDs and sample size consideration based on theoretical saturation and the nature of the study were informed by the previous studies [28,29]. Interviews were recorded, and major points were noted during the interview. 

### Data analysis

Thematic analysis was used to transcribe, summarize, and categorize all qualitative data, and the results were presented using texts, illustration quotes, and tables. Data from the questionnaire survey were analyzed using frequencies and association tests (significance statistics and strength statistics) in Statistical Package for the Social Sciences IBM^®^ (SPSS IBM 26 version, USA). Data on preference-related variables were analyzed using frequencies. Before proceeding with the analysis, the biotechnology awareness data were checked for internal consistency (KR-20 = 0.854). Scores for respondents’ awareness were computed and then binned or quantized into three groups: low awareness, moderate awareness, and high awareness. Cross-tabulation was carried out to obtain frequencies for awareness levels across categories for each sociodemographic characteristic. Furthermore, tests for the association using significance statistics (chi-square and likelihood ratio chi-square tests) and strength statistics (Cramer’s *V* and gamma tests) were also carried out. All quantitative results are presented using text and tables.

## Results

### Respondents’ sociodemographic characteristics and awareness levels

Sociodemographic characteristics and awareness levels of respondents toward reproductive biotechnologies are presented in Table 1. Out of 622 respondents, 416 (66.9%) were female and 206 (33.1%) were male. The majority (64.8%) of respondents had attended a primary level of education. A slightly higher number (43.1%) of respondents were 30–45 years old. Many (65.1%) respondents had a monthly income of K500 (ZMW500). The majority (65.9%) had less than 6 years of rearing experience. With regard to awareness, many (74.1%) respondents had a low level of awareness; the majority (55.8%) of them were female. The majority (52.6%) of the respondents were in the low awareness category with a primary level of education status. Furthermore, a slight majority (32.6%) of respondents had low awareness levels between 30 and 45 years of age. Across income status, many (51.0%) respondents had low awareness with a monthly income of less than K500 (ZMW500). On the other hand, those with less than 6 years of rearing experience and low awareness levels dominated (52.2%) the pig farming activity.

### Analysis of the association between awareness and sociodemographic characteristics

The results for biotechnology awareness and sociodemographic associations are presented in Table 2. Chi-square tests indicated a significant relationship between biotechnology awareness and gender predisposition (*p* < 0.001), income status (*p* < 0.01), and rearing experience (*p* < 0.001). There was no significant relationship (*p* > 0.05) between awareness and respondents’ age. The likelihood ratio chi-square test revealed a relationship (*p* < 0.001) between biotechnology awareness and education status. Also, gamma analysis revealed that biotechnology awareness significantly increased with an increase in the level of education (*p* < 0.001), income status (*p* < 0.01), and rearing experience (*p *< 0.001) of the respondents. There was a significantly strong relationship (*p *< 0.001) between biotechnology awareness and gender predisposition.

**Table 1.** Cross-tabulation of respondents’ sociodemographics and awareness levels toward biotechnologies.

Variable	Category	Awareness level			
		Low awareness	Moderate awareness	High awareness	Total
Gender	Male (% of total)	114 (18.3)	58 (9.3)	34 (5.5)	206 (33.1)
	Female (% of total)	347 (55.8)	55 (8.8)	14 (2.3)	416 (66.9)
	Total (% of total)	461 (74.1)	113 (18.2)	48 (7.7)	622 (100)
Education level	Uneducated (% of total)	53 (8.5)	8 (1.3)	1 (0.2)	62 (10.0)
	Primary (% of total)	327 (52.6)	56 (9.0)	20 (3.2)	403 (64.8)
	Secondary (% of total)	81 (13.0)	47 (7.5)	26 (4.2)	154 (24.7)
	Tertiary (% of total)	0 (0.0)	2 (0.3)	1 (0.2)	3 (0.5)
	Total (% of total)	461 (74.1)	113 (18.2)	48 (7.7)	622 (100)
Age	Below 30 years (% of total)	77 (12.4)	19 (3.0)	5 (0.8)	101 (16.2)
	30–45 years (% of total)	203 (32.6)	47 (7.6)	18 (2.9)	268 (43.1)
	Above 45 years (% of total)	181 (29.1)	47 (7.6)	25 (4.0)	253 (40.7)
	Total (% of total)	461 (74.1)	113 (18.2)	48 (7.7)	622 (100)
Income status	Below K500 (% of total)	317 (51.0)	66 (10.6)	22 (3.5)	405 (65.1)
	K500 to K2000 (% of total)	101 (16.2)	36 (5.8)	21 (3.4)	158 (25.4)
	Above K2000 (% of total)	43 (6.9)	11 (1.8)	5 (0.8)	59 (9.5)
	Total (% of total)	461 (74.1)	113 (18.2)	48 (7.7)	622 (100)
Years of experience	Below 6 years (% of total)	325 (52.2)	71 (11.4)	14 (2.3)	410 (65.9)
	6–10 years (% of total)	65 (10.4)	16 (2.6)	11 (1.8)	92 (14.8)
	Above 10 years (% of total)	71 (11.4)	26 (4.2)	23 (3.7)	120 (19.3)
	Total (% of total)	461 (74.1)	113 (18.2)	48 (7.7)	622 (100)

K1 = ZMW1, US$1 = ZMW16.8 (10/7/2021).

**Table 2.** Summary statistics for awareness across sociodemographic characteristics.

Variable	Test for awareness association				
	Significance statistic	df	*p*-value	Strength statistic	*p*-value
Gender	*χ*^2^ = 62.388	2	0.000^***^	φc = 0.317	0.000^***^
Education level	*G*^2^ = 60.112	6	0.000^***^	*γ* = 0.524	0.000^***^
Age	*χ*^2^ =3.374	4	0.497^*^	*γ* = 0.098	0.182^*^
Income status	*χ*^2^ = 14.958	4	0.005^**^	*γ* = 0.238	0.003^**^
Rearing experience	*χ*^2^ = 38.601	4	0.000^***^	*γ* = 0.362	0.000^***^

*** Statistically significant at *p *< 0.001.

** Statistically significant at *p *< 0.01.

* Not statistically significant at *p *> 0.05.

df = degrees of freedom, *χ*^2^ = chi-square, *G*^2 ^= Likelihood ratio chi-squared, φc = Cramer’s V, *γ* = gamma.

### The popularity of individual biotechnologies and the sources of information

With regard to information flow, 14.0% of the respondents acquired information about reproductive biotechnologies from fellow farmers; 11.1% of them acquired it from livestock officers; 4.8% got it from either school or through formal education; and 4.2% acquired it from televisions and radios; a total of 70.0% of the respondents did not give responses about their sources of information. It was also observed that some respondents acquired information from more than one source. Furthermore, among the reproductive biotechnologies respondents were aware of, AI was the most popular (29.3%) biotechnology by far (Table 3). 

### Pig rearing and traits of indigenous pigs preferred by traditional farmers

Out of 622 respondents, 339 (54.5%) valued indigenous pigs over exotic breeds (283; 45.5%). Results showing respondents’ reasons for or motivation for pig farming activity are presented in Table 4; the majority (56.9%) of them indicated an additional source of income as their motivation for rearing pigs. When asked about unique traits they prefer in indigenous pigs, seven traits were identified. Of these, 25.9% and 23.8% of the respondents reported disease resistance and fertility as their preferred traits, respectively. In addition, some respondents preferred more than one pig trait.

### Focus group discussions

To shed more light on the questionnaire survey and to obtain additional participants’ views on indigenous pigs, their preferred traits, and their biotechnology awareness, FGDs were conducted. Two big ideas came out of the FGDs: (1) what people thought in general about indigenous pig farming and (2) how aware people were of biotechnologies and what they thought their benefits were. 

### The general opinions about indigenous pig rearing

During the discussions, pigs were regarded as a crucial livestock species in the livelihoods of rural farmers. Generally, participants indicated that pigs helped them to reduce their poverty and poor livelihood situations. To this end, several roles played by pigs were identified: source of income; meat for home consumption; sociocultural roles (used for funeral rites and paying dowry); manure from fecal matter; and source of employment for both men and women. Some participants were quoted verbatim.

“*Ah, I sell pigs to take my children to school, buy food, blankets, and even fertilizer for my crops*” (Participant No. 6—FGD Lumbo, Gwembe District). 

“*Actually pigs help us to solve our financial needs and other problems. Ah, we also eat meat from our pigs, … so, they are very beneficial because we can even get manure from their dang or we use the money after selling pigs to buy fertilizer*” (Participant No. 2—FGD Mumba, Petauke District). 

The main motivations for rearing pigs were reportedly income generation and a source of meat for home consumption. For the former, participants sold indigenous pigs at prices ranging from ZMW250 to ZMW800, and exotic pigs such as the Large White fetched about ZMW3,500. In addition, two indigenous pig strains, namely Lusitu and Nsenga pigs, whose places of origin are Gwembe and Petauke districts, respectively, were mentioned. These pigs were highly valued over exotic breeds, citing their traits which participants cherished.

**Table 3.** Reproductive biotechnologies known by the respondents.

Variables	Category	Frequency(%)
Awareness of AI	No	440 (70.7%)
	Yes	182 (29.3%)
Awareness of semen evaluation	No	569 (91.5%)
	Yes	53 (8.5%)
Awareness of OI and OS	No	550 (88.4%)
	Yes	72 (11.6%)
Awareness of semen preservation	No	573 (92.1%)
	Yes	49 (7.9%)
Awareness of IVF and ET	No	586 (94.2%)
	Yes	36 (5.8%)
Awareness of heat detection methods	No	535 (86.0%)
	Yes	87 (14.0%)
Awareness of PD methods	No	568 (91.3%)
	Yes	54 (8.7%)

AI = artificial insemination; OI and OS = estrous induction and synchronization; IVF and ET = *in vitro* fertilization and embryo transfer; PD = pregnancy diagnosis.

**Table 4.** Motivation for pig farming and indigenous pig traits preferred by farmers.

Variables	Category	Frequency(%)
Motivation	Primary income	255 (41.0%)
	Additional income	354 (56.9%)
	Hobby or pet	8 (1.3%)
	Home consumption	129 (20.7%)
Trait preference	Disease resistance	161 (25.9%)
	Growth rate	29 (4.7%)
	Age at first mating	20 (3.2%)
	Good meat quality	17 (2.7%)
	Litter size	148 (23.8%)
	Foraging ability	123 (19.8%)
	Lusty or hardy	9 (1.4%)
	No answer	245 (39.4%)

“*The Nsenga pig is one of the breeds that increase in number fast, the only thing we need is that they should not continue to die because these pigs if we have them we can increase income at a faster rate*” (Participant No. 7—FGD Petauke, Petauke District). 

A plethora of preferred indigenous pig traits reported during FGDs are summarized, together with the reason(s) for their preference, in Table 5.

Participants desired to conserve the indigenous pigs because of their crucial contribution to their livelihoods; they raised several requests to support indigenous pig conservation. This study observed that participants were worried about indigenous pigs’ disappearance; they desired to see them conserved.

“*My opinions about indigenous pigs is that, they should be conserved and…may be, the new technologies can help to increase them faster because with these pigs we get meat, pay school fees, buy foods. …Even, they are disease resistant*” (Participant No. 3—FGD Kalindawaalo, Petauke District). 

“*My thought is that these pigs that are coming might finish our local pigs, so we need to keep our local breeds so that they don’t get finished*” (Participant No. 1—FGD Makuyu, Gwembe District). 

Furthermore, two potential approaches to conserving these breeds were identified, namely (1) the culture of giving a pig to a friend who does not have one and sharing piglets when they produce them, which was believed to promote in-situ conservation and (2) their opinion about reproductive biotechnologies like gamete preservation as a potential method of indigenous pig conservation (ex-situ). 

Many participants valued indigenous pigs for their various desirable traits, while some of them indicated that they were not comfortable with their small size compared to exotic breeds. They reasoned that small pigs do not fetch more money, yet they keep pigs primarily for income generation. 

“*We want these local pigs kept because they are good but the only challenge is that the breed is too small, and we think when we use the new methods, they will improve the size*” (Participant No. 2—FGD Lumbo, Gwembe District). 

“*The breed is so small, just that we need methods that can increase their size because they are too small, we don’t sale at a good price*” (Participant No. 5—Muyumbwe, Gwembe District).

### Participants’ awareness of biotechnologies and their perceived advantages

During the FGDs, participants mentioned several concerns about pig farming activity, namely diseases, small breed size, breed extinction threat, feed shortage, inbreeding, lack of sound breeding boars, delayed heat, and general reproductive insufficiency. Most of the people who took part were optimistic about how reproductive biotechnologies could help improve production and productivity and protect their native pigs. 

“*When these methods are embraced our pigs will continue*” (Participant No. 7—FGD Kalindawaalo, Petauke District). 

“*Our pigs are too small but using biotechnologies, we can change the breed and our livelihoods can change too*” (Participant No. 3—FGD Muyumbwe, Gwembe District). 

**Table 5.** Preferred traits of indigenous pig breeds among traditional pig farmers.

Pig trait	Reason(s) for preference of the trait
Disease resistance	Even when a disease like a swine fever comes, some local pigs survive. We do not even buy drugs they just get well on their own.
Litter size	Local pigs normally give many piglets, about 10–15, or even 18, but some can give a few like 4 or 5 piglets.
Meat quality	The meat of our pigs tastes good, even customers like buying these pigs. The demand is high.
Foraging ability	Since we do not have commercial feeds, we keep our local pigs on free-range, they eat on their own. The other thing, we do not know how to manage pigs properly so we keep them on free-range.
Hardy or lusty	These local pigs can survive in our bad or harsh conditions including high temperatures.
Age at first mating	These local pigs can grow very fast, they can get pregnant at around 5–8 months of age and produce for us piglets.

In this study, some participants had heard about certain biotechnologies used in animal reproduction; the majority did not know anything regarding the same. For those aware, they were able to mention some biotechnologies as well as their perceived benefits in animal production (Table 6). The most common biotechnology they knew about was AI, followed by heat detection, heat induction, and synchronization. 

“*I heard about AI, I also heard that there are ways of making the pig come on heat, I also heard about the embryo transfer. And also, the other one I know is that of making animals pregnant at the same time*” (Participant No. 4—FGD Makuyu, Gwembe District).

“*Synchronization and heat induction are also good because if the pig is delaying to be on heat, this method will help the pig to go in heat faster. Ah, and you know we keep pigs mainly for business, when they are induced, you have a lot of pigs in a year*” (Participant No. 7—FGD Kalindawaalo, Petauke District).

It is noteworthy that even if some participants knew about these biotechnologies, they largely lacked general knowledge about animal species where particular biotechnologies are applicable, how they are used, their efficacy, as well as practicability under local farming conditions. It was obvious that participants generally had positive perceptions about these technologies. However, they were skeptical about their practicability on indigenous pigs under the local farming conditions.

“*Me I heard of the AI not being used for the pig but the cow*” (Participant No. 5—FGD Mumba, Petauke District). 

“*Embryo transfer is good although am not sure if it is possible to have more than one piglet. Now, is it possible to make a lot of piglets at once through embryo transfer?*” (Participant No. 8—FGD Mukuyu, Gwembe District)*.*

## Discussion

Biotechnology adoption by farmers, particularly in African countries, remains minimal; one major factor that affects adoption is information acquisition or biotechnology awareness [18,30,31]. This is because people’s attitudes, including the decision to accept and/or adopt technology, change when they become aware of it [16,25,32]. In view of this, the observed dominance of respondents with low awareness presents a concern worthy of the attention of biotechnology policymakers and implementers; one cannot adopt biotechnology without getting to know about it [33]. Previously, it was confirmed that it is not uncommon to find substantial levels of nonexposure and nonawareness among smallholder farmers [16]. Given the current findings and the desire for biotechnology application, there is a need to raise biotechnology awareness level(s) in order to achieve widespread acceptance and adoption rate(s). Of note, agricultural extension is the vehicle through which information is delivered to farmers so that they are aware [32,34]. Ediset and Madarisa [17] reported that the extension method influences the rate of biotechnology adoption by farmers, considering the previous studies and the currently observed information sources, demonstrations, and farm and home visits will promote awareness.

Most of the respondents in this study were aware of AI than any other reproductive biotechnology; this was generally suggestive of its popularity. This is consistent with the previous reports, which indicated that AI biotechnology was more popular than other reproductive biotechnologies [8,13,14,30]. Perhaps, AI was popular because it is among the oldest biotechnologies globally used in animal production [14,15,30]. Nevertheless, even if AI-supporting biotechnologies were less popular than AI, their influence on AI’s success rate cannot be ignored [14,35]. Thus, efforts to raise farmer awareness of AI-supporting biotechnologies would also benefit the effort to modernize livestock production. Information acquisition, especially about AI-supporting biotechnologies, will promote farmers’ awareness and thus innovation adoption [31]. Furthermore, efforts to validate their practicability in the local rearing conditions through trials on indigenous pig genotypes will allay existing uncertainties as observed during FGDs.

**Table 6.** Participants’ awareness about biotechnologies and their perceived advantages.

Biotechnology heard of (aware of)	Perceived advantage or benefit(s)
AI	Many times, we do not have boars; therefore, it helps us to breed our sows. It can be used to breed sows even if the male died or is sick but you had stored its semen. With AI, you can reduce the breeding period between farrowing and next mating. AI can be used to improve our small breed.
Heat detection methods	They help a farmer to know when to breed the sow. They can also help us to predict the time a pig will be pregnant or when to perform AI. When you know that my pig is on heat, you will know what to do next.
Semen evaluation	It helps you to evaluate semen and screen the boar that is sick, and when sick you do not use its semen.
Heat induction and synchronization	When the pig is delaying coming on heat, you can use these methods to induce heat. You can use these methods to change the heat period to the time you want. It can also help to increase the number of piglets. It can also help you to know the time when pigs will go on heat.
Embryo transfer	This is also good because it can increase the number of piglets. It helps obtain piglets faster.
Preservation of semen	You can store semen and use it even if the boar died or is sick. It can also help use semen next time you want to do AI.
Pregnancy detection methods	They can be used to see if semen was “fruitful” (fertile) after AI. They help you to know and find means of caring for the pregnant sows. You will also know when the pig will farrow.

Sociodemographic characteristics affect exposure to biotechnologies, their perceived advantages, and respondents’ decisions to get information from extension workers [16,36]. As such, information about sociodemographic characteristics in relation to biotechnology awareness in this study will be crucial in guiding biotechnology policy and further research. This study revealed the dominance of female farmers in pig farming practice, which is similar to previous reports in Botswana and Sierra Leone, where 62% and 61.3% of the farmers, respectively, were female [37,38]. Although most respondents were female, their biotechnology awareness level was lower than that of males. It was previously confirmed that females are mostly preoccupied with domestic responsibilities; they have less time to interact with people knowledgeable about biotechnologies, unlike males, hence their lower awareness level than males [36,39]. Given female dominance, interventions aimed at increasing biotechnology acceptance and adoption rates must account for this knowledge gap; moreover, females play an important role in household food and economic security [5].

The dominance of respondents with a low level of education concurs with earlier findings in India [40]. It is plausible that many respondents were less educated because people with a lower education status do not get a permanent employment in the formal sector [8,38]. Incidentally, the less educated and the majority had lower biotechnology awareness. Previous studies confirmed that farmers with low education status have less access to agricultural information and the minimal capacity to process it [31]. The current study revealed a significant positive association between biotechnology awareness and education status. Llewellyn and Brown [16] explain that higher levels of education are associated with more active knowledge-seeking behavior, the ability to process information and appreciate biotechnology relevance. Hence, the stopgap measure in the current situation, where the majority are less educated, is establishing a robust extension system to improve the existing low awareness status. This is because extension service access counteracts the negative effect of a lack of formal education on biotechnology awareness and adoption rate [31].

Middle-aged respondents dominated the pig rearing activity. This was similar to the earlier study findings in India, where 60% of the farmers were in the middle-age (30–40 years) category [40]. It was confirmed that the involvement of young adults in livestock farming is critical due to their immense contribution to farmers’ livelihoods [41]. This notwithstanding, the current study did not reveal a relationship between biotechnology awareness and respondents’ age. This could be explained by the previous reports [36,39], which confirmed the independence of access to information and age, as well as a communication channel and/or choice of information source and age. So, it makes sense that future interventions involving biotechnology should be sensitive to all age groups.

The majority of respondents were low-income earners consistent with previous findings in India, with 85% of the farmers living under the poverty data line [40]. Such a scenario was previously associated with farmers’ held belief that pig farming activity is for the poor [8,20,37,38]. Even though reproductive biotechnologies improve pig production and can positively contribute to the economic situations of farmers [13], the observed low level of awareness, especially among low-income earners, may be a potential barrier to biotechnology adoption. Abigaba et al. [8] reported positive attitudes toward reproductive biotechnology among low-income farmers; however, the same study revealed a more positive attitude evaluation with higher income status. The present study revealed that increasing awareness levels were associated with higher income status, probably due to enhanced capacity for information access. This is in view of the previous report, which confirmed that rural farmers with a higher income level had better access to agricultural information than low-income earners [39]. Biotechnology awareness interventions mainly targeting low-income earners will be key; furthermore, FGDs revealed the crucial importance of pigs as a source of income.

Most respondents had reared pigs for less than 6 years. This finding was similar to the previous report in Nigeria [33]. Earlier reports attributed the shorter rearing experience to economic hardships, the increasing animal protein deficit plaguing the rural communities, and the recent campaigns to keep pigs as a livelihood strategy [2,8]. Although many respondents had less than 6 years of experience rearing pigs and had low awareness, it is plausible that interventions that promote continuity of the pig rearing activity will favor biotechnology awareness, given the positive association between awareness and the rearing experience. The observed positive association could be attributed to the cumulative information and/or knowledge about biotechnologies that farmers acquire over time during their rearing experience. Also, since many of the farmers who answered the survey got their information from other farmers, it makes sense to use farmers with a lot of experience raising animals to pass on information from farmer to farmer.

### Preference for indigenous pigs by traditional farmers

In light of the aspirations for livestock modernization, research scientists, biotechnology policy framers, and implementers must appreciate farmers’ choices for pig traits and their motivation for farming. This would enable them to better align farmers’ needs with the scientific and breeding policy agenda [2,20]. In short, production improvement programs may fail to yield the desired results due to a mismatch between the programs’ breeding objectives and farmers [19]. In this study, most of the respondents preferred indigenous pigs to exotic breeds; qualitative and quantitative findings indicated that farmers cherished these pigs because of their unique traits. Earlier studies reported similar reasons why farmers’ value indigenous pigs [20,37,40]. A total of seven indigenous pig traits were identified, largely identical to the previous reports in South Africa and Ghana [20,41]. Considering the previous studies [19,20], these traits may be categorized into adaptive and productive/performance traits. Accordingly, biotechnology-related approaches to improving pig production must consider both trait forms.

Notably, all seven identified traits are generally important for production; however, only three of these, namely disease resistance, litter size, and foraging ability, received the most attention. Thus, these must not be ignored during pig breed (biotechnology) policy formulation because they may affect compliance during the later stages, for example, at the policy implementation level. There is a need to conserve these traits or the pig genotypes that possess them rather than replace them with unsuitable exotics or crossbreed them without regard to these desirable traits. The most mentioned pig trait was disease resistance; a similar finding was reported previously [19,38]. It is suggested that any change to how pigs are raised should try to keep this trait or make it even stronger while reducing disease challenges [19].

The second most mentioned pig trait was litter size, also known as “fertility.” Our finding was similar to the earlier reports from Sierra Leone, which indicated that prolificacy was among the most preferred indigenous pig traits [38]. The FGDs reported a litter size of 4–15 piglets with an average of about 9.5. This was similar to the previous findings (9.1 ± 1.76 piglets) in South Africa and slightly higher (5.3–8.8 piglets) than that reported in West Africa [20,42]. Due to the economic value attached to pigs, farmers would have opted for the exotic pigs because they are more prolific, but their adaptability to temperature extremes is low [9]. Of note, cases of small litter size can be managed by improving production approaches, for example, the application of reproductive biotechnologies [20,37,38].

Foraging ability, the third most mentioned, was the other preferred pig trait; a similar finding was also previously reported by Madzimure et al. [20]. Preference for this trait was attributed to feeding shortages, a challenge that compelled farmers to practice a free-range production system. Similarly, Madzimure et al. [20] reported a preference for this trait, citing indigenous pigs’ ability to scavenge for feed consequent to feed scarcity. This trait is very crucial given the harsh rearing conditions consequent to climate change effects and the poor economic conditions of traditional pig farmers. Climate change affects food and water availability [9,43]. Unfortunately, many farmers may not afford to buy feed since the majority of them are low-income earners. 

Although fewer respondents mentioned the other traits, interventions aimed to increase production need not trivialize them. In terms of the physiological, breeding and/or production aspects and socioeconomic aspects, their consideration will be crucial. For example, indigenous pigs’ hardy nature, which was reported previously [20,37], is and will continue to be critical given the rearing conditions [9]. For the participants, these pigs can survive the harsh rearing conditions. In many sub-Saharan countries, the challenges of feed scarcity, temperature extremes, diseases, and other effects of climate change are a reality and will continue to negatively impact livestock, including pig production [9,43]. Thus, since the Lusitu and Nsenga pigs are reportedly hardy, it is logical that they are conserved given the worsening climate change effects. Reproductive biotechnologies (ex-situ), such as gamete and embryo cryopreservation, will be needed as an additional measure against potential future loss [14,30]. Also, even though these pigs are hardy, they still need good care for them to perform even better [20].

The other traits identified were growth rate and age at first mating, which were reported previously in South Africa and Kenya [20,44]. Similar to our FGD findings, preference for these traits was attributed to farmers’ desire to have pigs grow or multiply faster so they could sell and generate a higher income [20]. This study revealed an age at the first mating range of 5–8 months, which was similar to the 4.5–9 months (males) and 6–9 months (gilts) reported in Kenya [44]. Furthermore, despite their acceptable growth rate, the small size of these pigs remains a concern to the farmers, given that many rear pigs for income generation. Nevertheless, like other traits, improving husbandry practices will likely result in a more desirable performance [20].

It is noteworthy that both qualitative and quantitative findings revealed the source of income and meat for home consumption as the primary motivations for pig rearing; similarly, these were reported in Botswana and South Africa [37,41]. Hence, indigenous pigs remain crucial to the livelihoods of traditional farmers and may further improve their economic and nutritional security conditions if production increases. As such, the current study findings will benefit various stakeholders who strive to increase pig production through biotechnology applications. Moreover, the study objectives aligned with the national goals of reducing the animal protein deficit and poverty levels among Zambians [2,7]. By and large, livestock development interventions need to focus on increasing production and preserving the preferred indigenous pig traits, knowing that climate variability and its effects are a reality [14]. 

## Conclusion

Pigs are inarguably crucial to the livelihoods of traditional farmers in Zambia; they rear them for income generation and meat for home consumption. Respondents valued indigenous pigs because of their unique productive and adaptive traits. This study has shown that most respondents had low overall awareness levels and across many sociodemographic characteristics. The biotechnology awareness level was significantly associated with many sociodemographic variables. Respondents were aware of AI more than they were of any other reproductive biotechnologies. We recommend increased biotechnology awareness among farmers, primarily through establishing a robust livestock extension system; the focus should be on the relevant biotechnologies, sociodemographic characteristics, and information sources. Studies on indigenous pig biology and those aimed at exploring the feasibility of reproductive biotechnologies with these pigs are urgently needed, mindful of the cherished traits. At the policy level, planning and implementation must consider the traits of native pigs that are most valued. This study has some limitations. (1) The findings may not be generalizable to all types of pig farmers. (2) Reproductive biotechnologies were not used in indigenous pig production in the study area, resulting in a lack of exposure, which may have contributed to the observed low awareness. Thus, our findings may not be generalizable to commercial farmers who mainly rear exotic breeds. (3) The current respondents were primarily recruited from rural areas. Hence, the findings may not closely reflect the views of urban pig farmers. (4) Tool construction was carried out assuming that reproductive biotechnologies were not being applied. Thus, some questions on knowledge assessment were not included. Accordingly, future studies should comprehensively assess the awareness and knowledge of pig farmers; comparisons between rural and urban, as well as commercial and traditional rearing conditions will be interesting. Furthermore, this study sought to identify the preferred indigenous pig traits. However, future research may be required to rank the identified traits to guide intervention programs appropriately. 

## List of abbreviations

AI, artificial insemination; FGD, focus group discussion; ZMW, Zambian Kwacha.
